# Japanese Encephalitis Outbreak, India, 2005

**DOI:** 10.3201/eid1209.060200

**Published:** 2006-09

**Authors:** Manmohan Parida, Paban K. Dash, Nagesh K. Tripathi, Ambuj, Santhosh Sannarangaiah, Parag Saxena, Surekha Agarwal, Ajay K. Sahni, Sanjay P. Singh, Arvind K. Rathi, Rakesh Bhargava, Ajay Abhyankar, Shailendra K. Verma, Putcha V. Lakshmana Rao, Krishnamurthy Sekhar

**Affiliations:** * Defence Research and Development Establishment, Gwalior, India;; † Air Force Hospital, Gorakhpur, India;; ‡ B.R.D. Medical College, Gorakhpur, India

**Keywords:** JE, Serodiagnosis, Molecular Epidemiology, dispatch

## Abstract

An outbreak of viral encephalitis occurred in Gorakhpur, India, from July through November 2005. The etiologic agent was confirmed to be Japanese encephalitis virus by analyzing 326 acute-phase clinical specimens for virus-specific antibodies and viral RNA and by virus isolation. Phylogenetic analysis showed that these isolates belonged to genogroup 3.

An epidemic of viral encephalitis was reported from July through November 2005 in Gorakhpur, Uttar Pradesh, India. It was the longest and most severe epidemic in 3 decades; 5,737 persons were affected in 7 districts of eastern Uttar Pradesh, and 1,344 persons died (*1*). Japanese encephalitis virus (JEV) is the most common cause of childhood viral encephalitis in the world; it causes an estimated 50,000 cases and 10,000 deaths annually (*2**,**3*). JEV is endemic in the Gorakhpur and Basti divisions of eastern Uttar Pradesh. The geographic features of this region are conducive for the spread of JEV; an abundance of rice fields and a bowl-shaped landscape allow water to collect in pools. Heavy rains saturated the ground in 2005, which caused ideal breeding conditions for mosquitoes that transmit the virus from pigs to humans. In addition, high temperature and relative humidity provided a suitable environment for JEV transmission.

We report in-depth investigations of JEV-specific antibodies, virus isolation, and demonstration of viral RNA in 326 febrile patients with encephalitis symptoms who were admitted to B.R.D. Medical College, Gorakhpur. Further molecular epidemiologic studies were performed to establish the genetic relatedness of the viral strain associated with this epidemic.

## The Study

A total of 326 clinical samples (185 blood and 141 cerebrospinal fluid [CSF]) were collected from the 326 patients who had a diagnosis of encephalitis. Two sets of blood samples, with and without anticoagulant, were collected for virus isolation and serologic tests. All serum and CSF samples were screened for JEV-specific immunoglobulin M (IgM) and IgG by using an in-house dipstick ELISA that incorporated nitrocellulose as the solid phase. Purified viral antigen was obtained from culture supernatant of infected C_6/36_ cultures by sucrose density gradient ultra centrifugation (*4**–**6*). Results were confirmed by using an in-house IgM capture ELISA (*7*).

JE-specific RNA was detected by using the Access quick one-step reverse transcription (RT)–PCR kit (Promega, Madison, WI, USA) with the primer pairs JED3S: ATG CGC GGA TCC GAC AAA CTG GCC CTG AA (1839–1867) and JED3C: GGG GAA GCT TCG TGC TTC CAG CTT TGT CC (2193–2165) on the basis of the sequence in domain III of the E gene of strain JaOArS982 (*8*).

Virus isolation was attempted in C_6/36_ cells (*4*) from RT-PCR– and IgM-positive serum and CSF samples according to standard protocol (*5*). Double-stranded sequencing of domain III of the E gene of JEV was performed on an ABI 310 sequencer (Applied Biosystems, Foster City, CA, USA) with the BigDye Terminator cycle sequencing ready reaction kit. The phylogenetic tree was constructed with the neighbor-joining method with bootstrap analysis of 1,000 replicates with the MEGA version 2.1 program (*9*).

Rural populations between the ages of 3 months and 15 years were affected; almost 50% of children 6–10 years of age were affected, and 35% of children <5 years of age were affected. The epidemic affected boys and girls at a ratio of 1.9 to 1. The overall case-fatality ratio was 23%. Children dominated the case load because most adults in the area are immune to the virus. The trend of the epidemic showed that most cases were reported from the first to third weeks of October. Clinical history showed that all patients had fever (temperatures 38.5°C–40°C); prominent symptoms included severe headache, convulsions, and vomiting, leading to paralysis, coma, and death.

Analysis indicated an overall positivity of 50% of serum samples and 30% of CSF samples. The antibody profile of the serum samples showed 23% IgM, 19% IgG, and 7% both IgM and IgG positivity, compared with 26% IgM, 4% IgG, and 1% both IgM and IgG positivity in CSF samples. A total of 9% of CSF samples were positive for JEV-specific RNA (355-bp amplicon) as determined by RT-PCR. All these RT-PCR–positive CSF samples were also positive for IgM. None of the serum samples were positive by RT-PCR for viral RNA. Adding RT-PCR– and IgM-positive samples to C_6/36_ cells yielded 7 JEV isolates from IgM-positive CSF samples only, as confirmed by ELISA and RT-PCR. The antibody profile of the RT-PCR– and isolation-positive samples is depicted in Table 1.

**Table 1:** Antibody profile of RT-PCR– and virus isolation–positive samples, 2005 Japanese encephalitis virus outbreak in India*

Serial number	IgM	IgG	RT-PCR	Virus isolation
1	+	–	+	–
2	+	–	+	–
3	+	–	+	–
4	+	–	+	–
5	+	–	+	–
6	+	–	+	–
7	+	–	+	–
8	+	–	+	–
9	+	–	+	–
10	+	–	+	–
11	+	–	+	–
12	+	–	+	–
13	+	–	+	–
14	+	–	–	+
15	+	–	–	+
16	+	–	–	+
17	+	–	–	+
18	+	–	–	+
19	+	–	–	+
20	+	–	–	+

*RT, reverse transcription; Ig, immunoglobulin.

Further analysis of a 355-nucleotide sequence in domain III of the E gene of these isolates showed >95% homology with JEV on BLAST search. On comparison with 24 other geographically diverse JEV isolates (Table 2), all JEV isolates sequenced in this study were closely related (>99% homology). The isolates from this outbreak showed a nucleotide sequence identity of 95.6% and 94.6% with prototype JEV (Nakayama strain) and the first Indian JEV (isolated from Vellore in 1956), respectively. The dendrogram showed that the JEV isolates responsible for the 2005 Gorakhpur epidemic belong to genogroup 3 (G3) but form a cluster separate from earlier Indian isolates (Figure).

**Table 2:** Japanese encephalitis viruses compared for sequence analysis*

Sl no.	Virus ID no.	Year of sample collection	Country	Source	GenBank accession no.
1	G8924	1956	India	Mosquito	U70394
2	826309	1982a	India	Human brain	U70403
3	NA	1982b	India	Human brain	U03689
4	733913	1973	India	Human brain	Z34095
5	GP78	1978a	India	Human brain	AF075723
6	782219	1978b	India	Human brain	U70402
7	7812474	1978c	India	Human brain	U70387
8	P20778	1958	India	Human brain	Z34096
9	NO	1995	Australia	Human serum	L43566
10	SA14	1954	China	Mosquito	U14163
11	JKT7003	1981a	Indonesia	Mosquito	U70408
12	JKT9092	1981b	Indonesia	Mosquito	U70409
13	JKT5441	1981c	Indonesia	Mosquito	U70406
14	Nakayama	1935	Japan	Human brain	U03694
15	JaOH0566	1966	Japan	Human brain	AY029207
16	JaNAr0590	1990	Japan	Mosquito	AY427795
17	95-167	1995	Japan	Pig blood	AY377579
18	JaNAr0102	2002	Japan	Mosquito	AY377577
19	K91P55	1991	Korea	Mosquito	U34928
20	WTP-70-22	1970	Malaysia	Mosquito	U70421
21	691004	1969	Sri Lanka	Human brain	Z34097
22	86VN207	1986	Vietnam	Human brain	AY376461
23	89VN49	1989	Vietnam	Human brain	AY376462
24	02VN22	2002	Vietnam	Pig blood	AY376465
25	GP14†	2005	India	Human CSF	NS
26	GP48†	2005	India	Human CSF	NS
27	GP55†	2005	India	Human CSF	NS
28	GP67†	2005	India	Human CSF	NS
29	GP82†	2005	India	Human CSF	NS

*NA, not available; CSF, cerebrospinal fluid; NS, not submitted. †Sequenced in this study.

**Figure:**
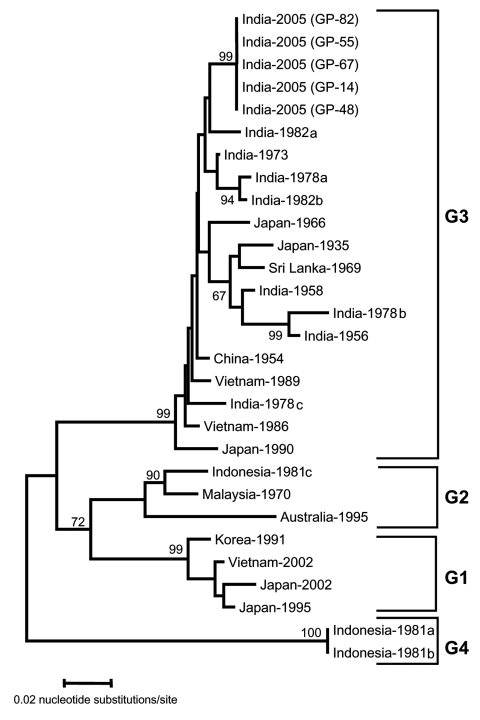
Sequence phylogeny based on partial E gene sequence of Japanese encephalitis virus isolates from the Gorakhpur epidemic, with reference to other Southeast Asian isolates. The tree was generated by neighbor-joining method. Each strain is abbreviated with the country of origin, followed by the year of isolation. Bootstrap values are indicated at the major branch points.

## Conclusions

The Gorakhpur district of Uttar Pradesh, which shares a border with Nepal and Bihar, has been experiencing periodic outbreaks of JEV since 1978. The virus cannot usually be isolated from clinical specimens, even with the best laboratory facilities, probably because of low levels of viremia and the rapid development of neutralizing antibodies. The diagnosis is therefore usually based on the presence of antibodies. The IgM capture ELISA for serum and CSF has become the accepted standard for diagnosing JEV.

The presence of only IgG antibodies in 19% of the patients indicated exposure to JEV infection in the past. This finding was expected because JEV is endemic to northern India, particularly Gorakhpur, and several large JEV epidemics have occurred in the past decade. In the present study, only 13 CSF samples (9%) were positive by RT-PCR. Seven virus isolates were obtained from IgM-positive CSF samples that did not yield RT-PCR amplicons before cultivation. Similar variations in virus detection and isolation have been reported (*10**,**11*); these findings underscore the sensitivity of cell culture systems for amplification of viable virus. Furthermore, the inability to detect genomic RNA or isolate virus from serum samples was striking and highlights the need for CSF sampling for both clinical diagnosis and epidemiologic studies.

We also investigated the molecular epidemiology of the outbreak by comparative sequence analysis of the isolates obtained in this study with reference strains of JEV. Domain III of the E gene was targeted for this purpose because this is the region under immune selective pressure, and it exhibits sufficiently rapid mutation to show evolutionary and epidemiologic relationships (*12**–**14*). We determined the partial sequence of these isolates directly from clinical samples without risk of altering the genome by passage in vitro. The dendrogram showed that the G3 of JEV is still circulating in India. However, compared with isolates from 1956 to 1988, recent isolates form a separate cluster. Frequent introduction of new virus genotypes through bird migration has led to shifts in circulating genotypes in neighboring Asian countries, including Japan, Vietnam, China, Korea, Sri Lanka, and Malaysia (*3**,**15*). Therefore, detailed and continuous epidemiologic surveillance is warranted to monitor the incursion and spread of JEV genotypes in India, which will allow effective control and management strategies to be undertaken at the earliest opportunity.
